# Pilot Investigation on the Metabolic Effects of *Cimicifuga racemosa* Extract Ze 450 and Voluntary Physical Activity in Female Rats

**DOI:** 10.3390/ijms27020977

**Published:** 2026-01-19

**Authors:** Elisabeth Habersatter, Tihomir Kostov, Nele Laing, Jürgen Drewe, Georg Boonen, Veronika Butterweck, Patrick Rene Diel

**Affiliations:** 1 Institute of Cardiology and Sports Medicine, German Sport University Cologne, 50933 Cologne, Germany; e.habersatter@dshs-koeln.de (E.H.); t.kostov@dshs-koeln.de (T.K.); nelejohanna.laing@stud.dshs-koeln.de (N.L.); 2 Medical Department, Max Zeller Soehne AG Switzerland, 8590 Romanshorn, Switzerland; jürgen.drewe@zellerag.ch (J.D.); georg.boonen@zellerag.ch (G.B.); veronika.butterweck@zellerag.ch (V.B.)

**Keywords:** *Cimicifuga racemosa*, Ze 450, physical inactivity, energy metabolism, visceral fat, leptin

## Abstract

*Cimicifuga racemosa* extracts, particularly the ethanolic extract Ze 450, are widely used to alleviate menopausal symptoms, such as hot flushes and excessive sweating. While their clinical efficacy is well established, the effects of these interventions on systemic energy metabolism remain unclear. This pilot study investigated the impact of Ze 450 on body composition, metabolic markers, and voluntary physical activity in non-ovariectomized female Wistar rats. Animals (N = 36) received Ze 450 at either 30 mg/kg or 130 mg/kg body weight, with or without access to voluntary wheel running over four weeks. Neither treatment influenced body weight gain or final body weight, indicating normal growth across all groups. Post-mortem analyses included visceral fat mass, serum cholesterol, and leptin levels. Both Ze 450 and running reduced visceral fat mass, adipocyte size, and circulating leptin levels, suggesting that they share overlapping mechanisms. Serum cholesterol was significantly lowered by running but remained unaffected by Ze 450, while liver weight and alanine aminotransferase activity were unchanged, confirming hepatic safety. Collectively, Ze 450 improved key metabolic parameters related to adiposity and appetite without affecting hepatic integrity, highlighting its potential as a safe, non-hormonal metabolic modulator complementary to physical activity.

## 1. Introduction

According to the World Health Organization, physical activity encompasses all bodily movements generated by skeletal muscles that require energy expenditure [1]. It is a vital determinant of health and well-being, whereas physical inactivity is a major contributor to the global rise in noncommunicable diseases (NCDs) and related mortality [2]. Individuals with insufficient activity levels are estimated to have a 20–30% higher risk of all-cause mortality compared with those who are physically active [2,3]. The burden of sedentary behavior on healthcare systems is profound and continues to grow [2]. Physical activity typically begins to decline during adolescence [4], coinciding with an increase in body weight [5]. Whether these trajectories persist through the transition into adulthood—a critical window for the development of obesity—remains uncertain [6,7]. Nevertheless, a robust body of evidence confirms that physical activity, particularly aerobic training, is effective in reducing body weight and fat mass in overweight and obese individuals [8,9,10]. Beyond these systemic effects, endurance training induces favorable molecular adaptations, including improved mitochondrial function and enhanced lipid oxidation capacity in skeletal muscle, which are key determinants of metabolic flexibility and energy homeostasis [11,12,13].

In addition to lifestyle interventions, plant-derived compounds are increasingly recognized for their ability to influence metabolic pathways relevant to energy balance and body weight regulation [14]. Among these, extract preparations of *Cimicifuga racemosa* (L.) Nutt. (Ranunculaceae), commonly known as black cohosh, are primarily used in clinical practice for the management of menopausal symptoms, which has led to extensive pharmacological characterization of these preparations [15,16].

In accordance with evaluations by the European Medicines Agency (EMA) and its Committee on Herbal Medicinal Products (HMPC), hepatotoxicity associated with *Cimicifuga racemosa* remains a potential but unconfirmed risk based on isolated case reports, while available clinical and non-clinical studies have not demonstrated intrinsic hepatotoxic effects [16,17]. As a precautionary measure, liver-related warnings have been included in the product information of *C. racemosa* medicinal products.

The ethanolic extract Ze 450 has emerged as a particularly well-characterized formulation of *C. racemosa*. Clinical and preclinical studies have suggested a role for Ze 450 in metabolic regulation, including effects on glucose and lipid metabolism and cellular energy homeostasis [18,19,20,21,22,23]. Mechanistic studies by Günther et al. demonstrated activation of AMP-activated protein kinase (AMPK) via Ze 450 in multiple cell types, including skeletal muscle, liver, adipocytes, and neurons, indicating a broad regulatory role in energy metabolism and insulin sensitivity [23]. In vitro investigations by Rabenau et al. further reported a Ze 450-induced shift in cellular energy metabolism, characterized by suppression of mitochondrial respiration and enhanced glycolysis, accompanied by increased cellular resilience to oxidative stress [20,21]. Consistent with these mechanistic findings, Moser et al. reported improvements in glycemic control and lipid metabolism in ob/ob mice treated with Ze 450 [19]. Collectively, these findings suggest that Ze 450 may impact specific metabolic processes related to energy balance and body weight control.

Against this background, the present pilot study was designed to investigate the effects of Ze 450 on energy balance, body composition, and voluntary physical activity in female rats. By using a non-ovariectomized model, this study aimed to assess potential metabolic effects of Ze 450 without experimental manipulation of ovarian function, thereby providing insight into its actions on energy homeostasis in a preclinical setting.

## 2. Results

### 2.1. Effects of Ze 450 and Physical Activity on Body Weight, Food Consumption, and Daily Energy Intake

**Figure 1:**
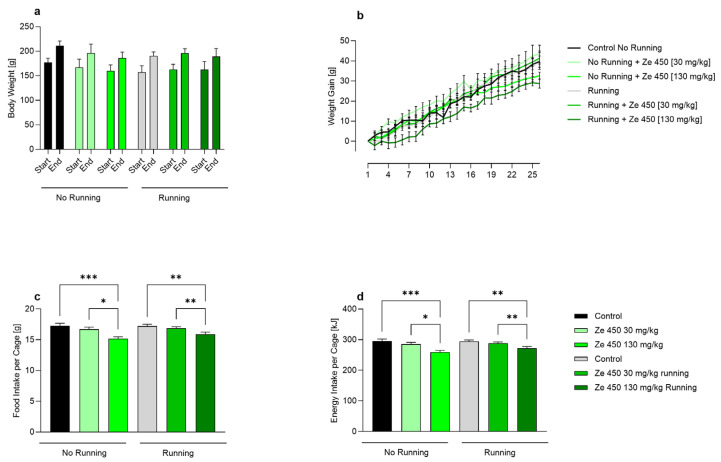
Effects of *Cimicifuga racemosa* extract Ze 450 and physical activity on body weight, food consumption, and energy intake in young female Wistar rats. (**a**) Body weight at the start and end of the experiment did not differ significantly between groups, irrespective of Ze 450 treatment or running condition. (**b**) Time course of weight gain throughout the study period showing no significant differences between groups. Food (**c**) and energy (**d**) intake per cage were significantly reduced via Ze 450 treatment in both sedentary and running animals. Data are presented as the mean ± SD, n = 6, * *p* < 0.05, ** *p* < 0.01, *** *p* < 0.001 versus the respective control group. Statistical analysis was performed using two-way analysis of variance (ANOVA) with the Holm–Sidak multiple comparisons test versus the respective control group.

To evaluate the effects of the *Cimicifuga racemosa* extract Ze 450 on weight gain and feeding behavior, body weight, food consumption, and energy intake were monitored throughout a 4-week intervention in non-ovariectomized female Wistar rats. At the beginning and end of the study, body weight did not differ significantly between groups (Figure 1a). Throughout the experimental period, both running and Ze 450 treatment at either 30 or 130 mg/kg had no measurable impact on final body weight compared with the respective controls. Similarly, the time course of body weight gain showed no statistically significant differences between treatment groups, regardless of exercise condition (Figure 1b). This indicates that neither Ze 450 nor voluntary running affected overall body weight development during the experimental period. In contrast, food intake was significantly reduced by Ze 450 administration (Figure 1c,d). Under sedentary conditions, both doses of Ze 450 led to a decrease in daily food intake per cage compared with control animals (*p* < 0.05 for 30 mg/kg; *p* < 0.001 for 130 mg/kg). A similar reduction was observed in the running groups, where Ze 450 treatment significantly lowered food consumption relative to running controls (*p* < 0.01). Cumulative food intake across the experimental period mirrored these findings, showing a dose-dependent reduction in both non-running and running animals treated with Ze 450 (*p* < 0.05–0.001). Overall, these data demonstrate that Ze 450 reduces food intake in a dose-dependent manner, independent of physical activity, without affecting body weight or overall weight gain.

### 2.2. Effects of Cimicifuga racemosa Extract Ze 450 on Visceral Fat Mass, Adipocyte Size, and Serum Leptin Levels

**Figure 2:**
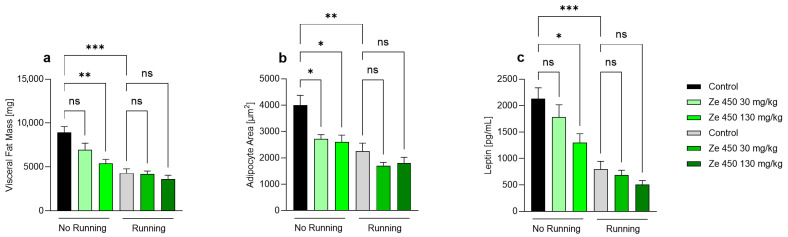
Effect of *Cimicifuga racemosa* extract Ze 450 on visceral fat mass, adipocyte size, and serum leptin in young female Wistar rats. (**a**) Visceral fat mass [mg]. (**b**) Adipocyte area [µm^2^] determined from histological sections. (**c**) Serum leptin concentrations [pg/mL]. Rats were assigned to six groups (n = 6 per group): Control (No Running, black), Control (Running, grey), No Running + Ze 450 [30 mg/kg] (light green), No Running + Ze 450 [130 mg/kg] (green), Running + Ze 450 [30 mg/kg] (light olive), and Running + Ze 450 [130 mg/kg] (dark olive). Ze 450 was incorporated into a phytoestrogen-free chow diet. Running groups had access to running wheels during the dark phase. Data are shown as the mean ± SD, n = 6 per group. Statistical analysis was performed using two-way ANOVA with the Holm–Sidak multiple comparisons test. * *p* < 0.05, ** *p* < 0.01, *** *p* < 0.001, ns = not significant.

Ze 450 treatment significantly reduced visceral adiposity in sedentary animals, while running alone had no additional effect (Figure 2a). In non-running groups, both Ze 450 doses decreased visceral fat mass compared with control animals (*p* < 0.01 for 30 mg/kg; *p* < 0.001 for 130 mg/kg). In running animals, visceral fat mass was lower overall, but no further reduction was observed upon Ze 450 treatment, indicating a ceiling effect of physical activity on fat mass reduction. Similarly, adipocyte size was significantly reduced by Ze 450 in sedentary animals (*p* < 0.05 for 30 mg/kg; *p* < 0.01 for 130 mg/kg), while running alone produced comparable effects (Figure 2b). The combination of Ze 450 treatment and running did not further decrease adipocyte area. Serum leptin levels closely reflected these morphological changes (Figure 2c). In sedentary animals, Ze 450 treatment markedly decreased leptin concentrations in a dose-dependent manner (*p* < 0.05 for 30 mg/kg; *p* < 0.001 for 130 mg/kg). Running alone also reduced leptin levels significantly, and no significant additive effects were observed when combined with Ze 450. Taken together, these results indicate that Ze 450 reduces visceral fat accumulation, adipocyte hypertrophy, and circulating leptin levels in a dose-dependent fashion. The effects of Ze 450 are comparable with those of voluntary running.

### 2.3. Effects of Ze 450 and Running on Plasma Cholesterol, Liver Weight, and Hepatic Enzyme Activity

Serum cholesterol levels were significantly reduced by voluntary running compared with sedentary controls (*p* < 0.05), whereas Ze 450 treatment alone did not produce a significant change (Table 1). In sedentary animals, mean cholesterol concentrations were 3.70 ± 0.33 mmol/L in controls and decreased slightly with Ze 450 treatment to 3.60 ± 0.20 mmol/L and 3.32 ± 0.27 mmol/L for the 30 mg/kg and 130 mg/kg doses, respectively. Running markedly lowered cholesterol levels to 3.03 ± 0.26 mmol/L, and this effect was not further enhanced via Ze 450 (3.00 ± 0.28 and 2.97 ± 0.18 mmol/L for 30 and 130 mg/kg, respectively). Liver weights did not differ significantly between groups, ranging from 8.5 ± 0.7 to 9.8 ± 0.6 g, indicating that neither Ze 450 treatment nor running influenced relative liver mass. Plasma alanine aminotransferase (ALT) activity, a marker of hepatic integrity, remained within the normal physiological range in all groups and showed no significant differences between treatments. Mean ALT values varied between 104.8 ± 17.7 and 132.0 ± 67.1 U/L. Collectively, these findings indicate that within the scope of this pilot study, Ze 450 did not produce detectable alterations in liver weight or plasma ALT levels, and that the cholesterol-lowering effect observed in this study is primarily attributable to physical activity rather than Ze 450 treatment.

### 2.4. Effects of Cimicifuga racemosa Extract Ze 450 on Voluntary Running Activity

**Figure 3:**
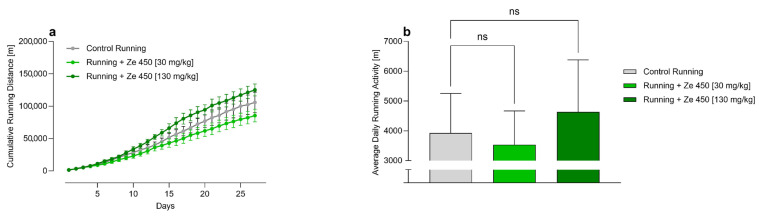
Effect of *Cimicifuga racemosa* extract Ze 450 on voluntary running activity in young female Wistar rats. (**a**) Cumulative running distance [m] over the 4-week intervention period. (**b**) Average daily running activity [m] during the intervention. Of the six experimental groups, only three were given access to running wheels and are shown here (n = 6 per group): Control (Running, grey), Running + Ze 450 [30 mg/kg] (light olive), and Running + Ze 450 [130 mg/kg] (dark olive). Ze 450 was incorporated into phytoestrogen-free chow, which was provided ad libitum. Running wheels were available during the dark phase. Data are shown as mean ± SD, n = 6 per group. Statistical analysis was performed using one-way ANOVA with the Holm–Sidak multiple comparisons test (ns = not significant).

**Figure A1:**
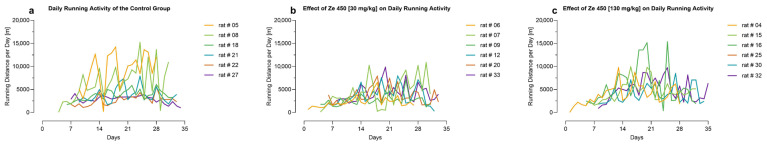
Individual daily running distances in young female Wistar rats. (**a**) Daily running activity in the control group (physical activity), n = 6. (**b**) Daily running activity in rats receiving *Cimicifuga racemosa* extract Ze 450 [30 mg/kg], n = 6. (**c**) Daily running activity in rats receiving Ze 450 [130 mg/kg], n = 6. Each line represents an individual animal monitored for 35 days. Ze 450 was incorporated into a phytoestrogen-free chow diet and provided ad libitum. Running wheels were available during the dark phase.

To evaluate whether *Cimicifuga racemosa* extract Ze 450 influences voluntary running activity, running distances were continuously monitored in rats with access to running wheels. The cumulative running distance over the 4-week intervention period in all physically active groups is shown in Figure 3a. While both Ze 450-treated groups followed a similar upward trajectory, the Running + Ze 450 [130 mg/kg] group consistently exhibited the highest cumulative running distance across the entire study period. However, this difference did not reach statistical significance, likely due to the small sample size (n = 6) and relatively high inter-individual variability in voluntary running behavior (Figure 3a). Similarly, average daily running activity did not differ significantly between groups (Figure 3b). Median daily running distances ranged between approximately 3000 and 5000 m per day, with high interindividual variability but no consistent effect of Ze 450 treatment. To further characterize individual variability in spontaneous activity, the daily running distances of individual animals are presented in Figure A1 (Appendix A). Considerable inter-individual differences were observed within all groups, with peak daily running distances occasionally exceeding 15,000–20,000 m. Notably, several animals in the Running + Ze 450 [130 mg/kg] group exhibited consistently higher peak running activity compared with both the Running Control and Running + Ze 450 [30 mg/kg] groups. This individual variability likely contributed to the lack of statistical significance observed for cumulative running distances, despite visible trends. In addition, daily wheel-running patterns showed the expected estrous-cycle-related oscillations in all groups, and no irregularities indicative of disrupted cyclicity were observed (Figure A1, Appendix A). These findings suggest a dose-dependent trend toward increased voluntary physical activity with Ze 450, with the higher dose potentially enhancing motivational or metabolic pathways involved in locomotor behavior. However, larger studies with greater statistical power will be required to confirm these observations.

## 3. Discussion

This study investigated the combined effects of *Cimicifuga racemosa* extract Ze 450 and voluntary running activity on energy metabolism, body composition, appetite regulation, cholesterol levels, and spontaneous physical activity in non-ovariectomized female Wistar rats. This model allowed us to assess Ze 450’s metabolic activity under physiological hormonal conditions, independent of experimentally induced estrogen deficiency. As expected in growing animals, all groups displayed steady physiological weight gain throughout the 4-week study. Neither Ze 450 nor running activity impaired normal growth, and no differences in final body weight or weight gain were observed between groups, indicating that Ze 450 does not interfere with energy balance under physiological conditions.

Importantly, sedentary behavior is known to promote adiposity [24], and in the present study, physical inactivity (no running) was associated with a marked increase in visceral fat mass. Ze 450 treatment was associated with a significant reduction in daily and cumulative food intake, which coincided with reduced visceral fat mass. However, changes in adiposity likely reflect the integrated contributions of both energy intake and energy expenditure rather than a single dominant mechanism. Both Ze 450 treatment and voluntary physical activity were associated with reduced adipocyte size and lower circulating leptin levels. Given that leptin is a sensitive biomarker of adipose tissue mass [25], the observed reductions in circulating leptin are best interpreted as secondary to decreased visceral fat mass rather than indicative of a direct effect of Ze 450 on leptin synthesis.

Ze 450 influenced energy intake in a condition-dependent manner. Reductions in food intake were most pronounced under sedentary conditions, whereas this effect was attenuated in physically active animals, likely reflecting compensatory increases in energy demand associated with voluntary running activity. This pattern suggests that Ze 450 may modulate energy intake in response to metabolic context rather than inducing a generalized suppression of appetite. The concomitant reductions in visceral fat mass and adipocyte size point toward potentially favorable effects on energy homeostasis, which should be interpreted cautiously within the exploratory framework of this pilot study. Notably, the preservation of normal body-weight gain despite reduced caloric intake suggests adaptive adjustments in energy balance that warrant further mechanistic investigation regarding metabolic efficiency. Accordingly, this interpretation remains exploratory and requires confirmation in future studies.

With respect to hepatic safety, Ze 450 administration for 4 weeks did not alter liver weight or plasma ALT activity at either tested dose, indicating no detectable adverse effects on hepatic parameters within the study period. Histopathological analyses were not performed. Therefore, conclusions regarding long-term hepatic safety cannot be drawn. These findings are consistent with previous clinical and non-clinical reports indicating a generally favorable hepatic safety profile of *Cimicifuga racemosa* extracts [17].

Total cholesterol levels were reduced by voluntary running but not by Ze 450 treatment alone, suggesting that the cholesterol-lowering effect observed in this study is primarily attributable to increased physical activity. A modest, non-significant downward trend in serum cholesterol was observed with increasing Ze 450 dose, which may indicate a subtle lipid-modulatory action that becomes more apparent under conditions of elevated metabolic demand. These results align with prior evidence showing that Ze 450 influences mitochondrial energy metabolism and activates AMPK, a key regulator of lipid oxidation and cholesterol homeostasis [20,21,22,23]. However, the present data do not allow definitive conclusions regarding cholesterol regulation by Ze 450 in isolation.

Ze 450 did not significantly alter voluntary locomotor behavior, although a trend toward increased running activity was observed at the higher dose. All groups exhibited the expected progressive increase in running distance over time, reflecting normal adaptation to wheel access. While rats receiving 130 mg/kg Ze 450 displayed the highest overall running distances, these differences did not reach statistical significance, likely due to inter-individual variability and limited sample size. Thus, any potential influence of Ze 450 on spontaneous physical activity should be considered exploratory. Despite modest differences in running behavior, voluntary exercise produced a robust reduction in visceral fat mass without affecting total body weight, consistent with established physiological adaptations whereby endurance-type activity preferentially reduces visceral adiposity without necessarily altering total body weight [26,27,28].

Although estrous cyclicity was not directly assessed by vaginal cytology, the observed wheel-running patterns were compatible with normal estrous-related fluctuations described in female rats [29,30,31]. Because wheel running reflects a complex motivated behavior influenced by multiple physiological factors [32], these observations should be regarded as indirect indicators rather than definitive assessments of estrous function. Importantly, no evidence suggested a disruption of normal activity rhythms, supporting the interpretation that Ze 450 does not adversely affect hormonally modulated behavioral dynamics.

Several preclinical studies have demonstrated the metabolic effects of Ze 450 involving mitochondrial function and AMPK signaling [19,20,21,23]. Rabenau et al. [20,21] reported that Ze 450 inhibits mitochondrial complex I activity, shifting energy metabolism toward glycolysis via hypoxia-inducible factor-1α (HIF1α) and cMyc (Myc proto-oncogene protein) activation, thereby reducing reactive oxygen species (ROS) production and promoting mitochondrial resilience. Günther et al. [23] confirmed that Ze 450 activates AMPK, a central regulator of cellular energy homeostasis, across multiple tissues, indicating that Ze 450 can modulate cellular energy metabolism in a manner comparable with established metabolic modulators, such as metformin. Moser et al. [19] further demonstrated that Ze 450 improves glucose tolerance, lowers triglycerides, and reduces hyperglycemia in obese diabetic mice. Together, these findings provide a mechanistic framework consistent with the reductions in visceral fat mass and adipocyte size observed in the present study while remaining distinct from estrogen-mediated pathways.

This pilot study also has limitations. The sample size was relatively small, potentially limiting the statistical power. Body composition was assessed indirectly rather than via whole-body imaging, and no molecular analyses were performed to directly confirm pathway activation. Additionally, this study was conducted in young, growing animals, which limits its generalizability to older or metabolically compromised populations. Nevertheless, as an exploratory investigation, these findings provide foundational evidence for estrogen-independent metabolic effects of Ze 450 and inform the design of future mechanistic and translational studies.

## 4. Materials and Methods

### 4.1. Animals and Experimental Design

A total of 36 female Wistar rats (5–6 weeks old; Charles River Laboratories, Wilmington, MA, USA) were included in the study. Prior to the intervention, all animals underwent a 7–10-day acclimatization period under standardized environmental conditions: 22 ± 2 °C ambient temperature and a 12-h light/dark cycle (lights on at 7:00 AM and off at 7:00 PM). Animals had ad libitum access to water and a phytoestrogen-free chow diet throughout the study. Following acclimatization, animals were randomly assigned (n = 6 per group) to one of six experimental groups: (1) no physical activity, (2) physical activity (Running), (3) No Running + Ze 450 [30 mg/kg], (4) No Running + Ze 450 [130 mg/kg], (5) Running + Ze 450 [30 mg/kg], and (6) Running + Ze 450 [130 mg/kg]. Group allocation was performed using a randomization procedure, and each rat was assigned an individual identification number. During the study, animals were housed in groups of three per cage (Sealsafe^®^ NEXT, Tecniplast S.p.A., Buguggiate, Italy), except during physical activity sessions. Rats in activity groups were individually placed in cages equipped with attached running wheels (a custom-built apparatus constructed at the German Sport University, Cologne, Germany) during the dark phase and returned to their home cages the following morning. Running distance was recorded daily. Inactivity groups remained in standard cages without wheel access. At the end of the intervention, animals were euthanized, and tissue and blood samples were collected for subsequent biochemical and histological analyses.

All procedures were conducted according to national and institutional guidelines for the care and use of laboratory animals. The study protocol was approved by the institutional ethics committee (protocol number: [81-02.04.2022.A306]).

### 4.2. Preparation of Diet and Ze 450 Administration

The experimental diet was based on a phytoestrogen-free rodent chow (Ssniff, V1554), formulated from a balanced blend of wheat, corn, barley, and essential nutrients (Sniff Spezialdiäten GmbH, Soest, Germany). The macronutrient profile consisted of 19.1% crude protein, 10% crude fat, 3.4% crude fiber, and 55.1% nitrogen-free extract substances, providing a nutritionally adequate baseline for the intervention. The ethanolic (60% *v*/*v*) *Cimicifuga racemosa* dry extract Ze 450 (drug-to-extract ratio, 4.5–8.5:1) was obtained from Max Zeller Söhne AG (Romanshorn, Switzerland; Batch No. 210301). The extract was produced from dried roots and rhizomes of *C. racemosa* and contained 8% triterpene glycosides. Ze 450 is a standardized, commercially manufactured extract with a reproducible phytochemical profile and is produced under controlled extraction conditions in compliance with HMPC requirements [17]. The chemical composition of Ze 450 has been described previously [33]. Ze 450 was incorporated into the chow at two concentrations to achieve target daily doses of 30 and 130 mg/kg body weight (BW). These doses were selected based on previously published in vivo studies in rodents, in which *Cimicifuga racemosa* extracts produced metabolic and endocrine effects within comparable dosage ranges [34]. Thus, the selected doses reflect pharmacologically active but well-tolerated levels.

The chow was prepared in bulk to cover the entire four-week intervention period, including an additional margin to accommodate sample collection needs and potential food loss. The formulation remained constant throughout the study, and Ze 450 concentrations were not adjusted to account for changes in body weight. Consequently, animals—initially weighing slightly below the target range—likely received a slightly higher per-kilogram dose during the early phase of the experiment. This fixed-dose design ensured consistent exposure based on an estimated average weight, allowing for controlled comparisons between groups. 

### 4.3. Monitoring of Body Weight, Food Intake, and Physical Activity

Body weight was measured daily in the morning using an electronic balance (FA-1500-2, Faust Laborbedarf AG, Schaffhausen, Switzerland). Food intake was recorded each day by weighing the amount of chow provided and subtracting the remaining uneaten portion the following morning. For group-housed animals, food intake was assessed per cage and subsequently normalized to obtain an estimated intake per animal. Fresh chow was provided daily to ensure continuous ad libitum access. Physical activity was assessed by monitoring voluntary wheel running. Rats assigned to activity groups were transferred into individual cages equipped with running wheels at the onset of the dark phase, corresponding to their natural active period, as rats are nocturnal and perform the vast majority of voluntary running during the dark cycle. Running wheels remained available throughout this period. Animals were returned to their original group housing the following morning. Running distance (m) was recorded daily using a wheel-tracking system (a custom-built apparatus, German Sport University Cologne).

### 4.4. Sample Collection and Tissue Dissection

#### 4.4.1. Blood Collection

At the end of the four-week intervention period, animals were euthanized using a CO_2_ unit (Typ: GDU-2017, Zupromed GmbH, Cologne, Germany), followed by decapitation to ensure complete exsanguination. Immediately post-mortem, animals were held upside down over 2 mL Eppendorf tubes to allow for blood collection by gravity without anticoagulants. Collected blood samples were allowed to clot at room temperature and subsequently centrifuged at 2500× *g* for 10 min at 4 °C using a benchtop centrifuge (Centrifuge 5417R, Eppendorf, Hamburg, Germany). To prevent hemolysis, serum samples were refrigerated at 4 °C only after centrifugation and then stored at −20 °C until further analysis.

#### 4.4.2. Tissue Dissection and Preparation

Tissue weight was determined immediately after dissection using a precision balance (Kern 430-21, Kern & Sohn GmbH, Balingen, Germany). Visceral adiposity was quantified by dissecting and weighing the retroperitoneal, parametrial, and mesenterial fat pads immediately after sacrifice. These depots together represent a standard method for assessing visceral fat mass in female rodent models. Visceral fat tissue was then split into two portions, with one part frozen in liquid nitrogen for biochemical analyses and the other fixed in 4% paraformaldehyde (PFA; Merck Schuchardt OHG, Hohenbrunn, Germany) and subsequently paraffin-embedded for histological examination (adipocyte size measurement). Blocks were stored at room temperature and transferred to 4 °C (refrigerator) overnight prior to sectioning. For adipocyte size determination, paraffin-embedded visceral fat samples were cut into 10 µm sections using a microtome and stained with hematoxylin and eosin (H&E). The stained sections were visualized using a light microscope (Axiophot; Carl Zeiss AG, Oberkochen, Germany) equipped with a camera and JENOPTIK GRYPHAX^®^ software (version 2.2.0.1234, JENOPTIK AG, Jena, Germany). From each sample, overview images were taken at 2.5× magnification, followed by at least 15 images at 20× magnification. Adipocyte size was quantified manually using ImageJ software (Version 1.54d; NIH, Bethesda, MD, USA) by outlining 150 individual adipocytes per animal and calculating their cross-sectional area. Measurements were performed in a blinded manner, and data were exported to Microsoft Excel (Microsoft Corporation, Redmond, WA, USA) for further analysis.

Liver tissue was frozen in liquid nitrogen and stored at −80 °C for biochemical analysis.

### 4.5. Biochemical Analyses

#### 4.5.1. Serum Lipid Profile and Liver Markers

Serum concentrations of total cholesterol and alanine aminotransferase (ALT) were determined by Laboklin GmbH & Co., KG (Bad Kissingen, Germany), a certified commercial laboratory. Analyses were performed using validated standard enzymatic methods; additional kit specifications are available upon request.

#### 4.5.2. Leptin Quantification

Serum leptin concentrations were measured in-house using a commercially available enzyme-linked immunosorbent assay (ELISA) kit (Mouse & Rat Leptin Quantikine ELISA Kit, R&D Systems, Minneapolis, MN, USA). Serum samples were diluted 1:10 in accordance with the manufacturer’s protocol. All assays were performed in duplicate, and absorbance was measured using a Multiskan™ FC microplate reader (Thermo Fisher Scientific, Waltham, MA, USA). Final concentrations were calculated based on standard curves provided in the kit.

### 4.6. Verification of Ze 450 Incorporation into Diet

To verify the recovery and homogenous incorporation of Ze 450 into the chow pellets, selected triterpene glycosides characteristic of *Cimicifuga racemosa* extract—namely, 23-epi-26-deoxyactein, actein, cimiracemoside A-xyloside, and cimiracemoside A-arabinoside—were quantitatively analyzed. Feed samples corresponding to both dosing regimens (30 and 130 mg/kg body weight) were extracted and analyzed via HPLC-MS using a Synapt UPLC system equipped with a time-of-flight mass spectrometer (MS TOF) (Waters Corporation, Milford, MA, USA), following an adapted version of a validated method (PV-2562, Max Zeller Soehne AG, Romanshorn, Switzerland). Detection was achieved using selected ion recording (SIR) channels specific to each compound. Sample extraction involved methanol-based ultrasonic treatment, and quantification was carried out with external calibration and internal standardization (α-hederin). The measured concentrations of marker compounds in both chow formulations (30 and 130 mg/kg) were compared with the known composition of the Ze 450 reference extract (Batch No. 210301). Mean recovery rates relative to the reference extract were 103.6% and 103.9% for the low- and high-dose feed samples, respectively, indicating excellent recovery and stability of the incorporated triterpenes across all marker compounds

### 4.7. Statistical Analysis

Data were analyzed using GraphPad Prism 10 (GraphPad Software, San Diego, CA, USA). Results are presented as mean ± standard deviation (SD). In most analyses, group differences were assessed using two-way analysis of variance (ANOVA) to evaluate the main effects of Ze 450 treatment, physical activity, and their interaction. When significant main or interaction effects were detected, Holm–Sidak’s post hoc multiple comparisons test was applied for pairwise group comparisons. For Figure 3, data were analyzed using one-way ANOVA followed by Holm–Sidak’s multiple comparisons test to compare running activity between groups. A *p*-value < 0.05 was considered statistically significant.

## 5. Conclusions

The findings of our study suggest that Ze 450 may serve as a safe and effective metabolic modulator. In conditions characterized by physical inactivity, visceral adiposity, and metabolic dysregulation, it may support and enhance the effects of lifestyle interventions that focus on nutrition and physical activity. However, given the exploratory nature of this pilot study, larger studies with increased statistical power are warranted to confirm and expand these preliminary observations. Future investigations should specifically explore the metabolic actions of Ze 450 in established models of metabolic dysfunction, such as high-fat-diet-induced obesity, which more closely reflect the pathophysiology of metabolic syndrome and menopausal adiposity in humans. In addition, mechanistic studies at the molecular level will be essential. Finally, clinical trials are needed to assess the translational relevance, safety, and therapeutic potential of Ze 450 for metabolic disorders in postmenopausal women or other high-risk populations.

## Figures and Tables

**Table 1:** Effects of *Cimicifuga racemosa* extract Ze 450 and running activity on cholesterol levels, liver weight, and ALT levels in young female Wistar rats.

Group	Cholesterol [mmol/L] Mean ± SD	Liver Weight [g] Mean ± SD	ALT [U/L] Mean ± SD
Control No Running	3.70 ± 0.33	9.79 ± 0.64	111.10 ± 34.10
No Running + Ze 450 (30 mg/kg BW)	3.60 ± 0.20	9.06 ± 1.26	104.77 ± 17.73
No Running + Ze 450 (130 mg/kg BW)	3.32 ± 0.27	8.51 ± 0.67	109.05 ± 18.87
Running	3.03 ± 0.26 *	9.51 ± 0.76	121.40 ± 15.98
Running + Ze 450 (30 mg/kg BW)	3.00 ± 0.28	8.99 ± 0.58	132.00 ± 67.14
Running + Ze 450 (130 mg/kg BW)	2.97 ± 0.18	8.88 ± 1.02	111.30 ± 23.67

ALT—alanine aminotransferase, BW—body weight. Ze 450 was administered via chow at either 30 or 130 mg/kg body weight/day. Running activity was enabled via voluntary wheel running during the dark phase. Data are shown as mean ± SD, n = 6 per group. Statistical analysis was performed using two-way ANOVA with the Holm–Sidak multiple comparisons test. * *p* < 0.05.

## Data Availability

The data presented in this study are available upon request.

## Abbreviations

The following abbreviations are used in this manuscript:

AMPK	AMP-activated protein kinase
ANOVA	Analysis of variance
ALT	Alanine aminotransferase
BNO 1055	Specific *Cimicifuga racemosa* extract (standardized product designation)
BW	Body weight
cMyc	Myc proto-oncogene protein
CO_2_	Carbon dioxide
CT	Computed tomography
ELISA	Enzyme-linked immunosorbent assay
EMA	European Medicines Agency
H&E	Hematoxylin and eosin
HIF-1 α	Hypoxia-inducible factor-1α
HPLC-MS	High-performance liquid chromatography–mass spectrometry
HMPC	Committee on Herbal Medicinal Products
MS TOF	Mass spectrometry–time of flight
NCD	Noncommunicable disease
*ob/ob* mice	Genetically obese mice with leptin deficiency (*obese/obese*)
PFA	Paraformaldehyde
ROS	Reactive oxygen species
SD	Standard deviation
SIR	Selected ion recording
UPLC	Ultra-performance liquid chromatography
*v/v*	Volume per volume
Ze 450	Ethanolic extract of *Cimicifuga racemosa*
